# Epithelial-to-Mesenchymal Transition in Pancreatic Adenocarcinoma

**DOI:** 10.1100/tsw.2010.183

**Published:** 2010-10-01

**Authors:** Carla Cano, Yoshiharu Motoo, Juan L. Iovanna

**Affiliations:** ^1^ INSERM U624, “Cell Stress” Biology of Pancreas Stress Laboratory, Marseille, France; ^2^ Department of Medical Oncology, Kanazawa Medical University, Ishikawa, Japan

**Keywords:** cancer, pancreas, epithelial-to-mesenchymal transition, TGFbeta, metastasis

## Abstract

Epithelial to mesenchymal transition (EMT) is a physiologic process that allows morphological and genetic changes of carcinoma cells from an epithelial to a mesenchymal phenotype, which is the basis of the high metastatic potential of pancreatic cancer cells. EMT is triggered by various tumor microenvironmental factors, including cytokines, growth factors, and chemotherapeutic agents. This review summarizes the state-of-the-art knowledge on the molecular mechanisms that support pancreatic cancer EMT and the evidences that support its involvement in invasiveness/aggressiveness, and the drug resistance of pancreatic cancer cells.

## INTRODUCTION

Epithelial-to-mesenchymal transition (EMT) is the collection of events that allows the conversion of adherent epithelial cells, tightly bound to each other within an organized tissue, into independent fibroblastic cells possessing migratory properties and the ability to invade the extracellular matrix. EMT was first described in the early 1980s because of its pivotal role during embryonic development[1] and later because of its implication in the physiological response to injury. In the early 1990s, EMT attracted the attention of cancer researchers after the discovery of its strong association with growth, invasion, and metastasis of cancer cells[2,3]. Indeed, tumor cells convert from low- to high-grade malignancy partly through EMT[4,5]. EMT-related molecular pathways have been extensively investigated thereafter, and various genes and molecules have been identified as important factors in EMT of cancer cells[5,6].

Pancreatic adenocarcinoma is one of the most deadly cancers. As a matter of fact, today almost every patient that is diagnosed with this disease will die of it. The dismal prognosis of pancreatic cancer is mainly due to its high metastatic potential, the late manifestation of its symptoms, and its strong chemoresistance[7]. During the last years, substantial literature has documented the pathogenesis of this cancer and has led to the conclusion that pancreatic ductal epithelial cells are the original cells of this malignancy. Moreover, a great help to the understanding of pancreatic adenocarcinoma has come from the development and analysis of mutant mice that very closely reproduce the human disease. Both the human observations and the targeting of EMT-related pathways in murine models have shown the crucial role of EMT in the aggressiveness and lethality of pancreatic cancer. Since the general knowledge of

EMT was already deeply reviewed elsewhere[6], in this review, after overviewing basic EMT concepts, we would like to focus on the recent findings on EMT of pancreatic cancer cells.

## EMT BASICS

During EMT, the polarized and basal membrane–anchored epithelial cell undergoes a number of biochemical changes in order to acquire a mesenchymal fibroblastoid phenotype. In addition to an enhanced migration, matrix-remodeling capacity, and resistance to apoptosis, the resulting mesenchymal phenotype is characterized by several molecular and morphologic transformations. The hallmarks of EMT *in vitro* and *in vivo* include the acquisition of a spindle-like/fibroblastic morphology, the up-regulation of mesenchymal markers (i.e., S100A4, N-cadherin, vimentin, α-smooth muscle actin) and extracellular matrix components (collagens α1 and α2), the down-regulation of epithelial cell surface markers and cytoskeleton components (i.e., E-cadherin, ZO-1, claudins, occludins, cytokeratins), and the up-regulation and/or nuclear translocation of specific transcription factors (i.e., Snail, Slug, ZEB1/2, Twist1/2[8,9]). Modulation of these biomarkers is of course triggered by molecules naturally present in the tumor microenvironment (i.e., TGFβs, BMPs, VEGF, HGF), and also by stressful conditions produced during tumor growth or anticancer therapies. Most of these biochemical changes have been observed in pancreatic cancer cells and common inducers of EMT play a crucial role in pancreatic adenocarcinoma development.

## EMT-TRIGGERING FACTORS IN PANCREATIC CANCER

### Transforming Growth Factor (TGF)-β

TGFβ isoforms (TGFβ1, TGFβ2, and TGFβ3) belong to a large superfamily of structurally related growth factors with pleiotropic biological functions that includes the bone morphogenetic proteins (BMPs), activins, growth and differentiation factors (GDFs), and Nodal. The TGFβ, through binding to their receptors (TβRI, TβRII, and TβRIII/betaglycan), transduce a canonical downstream signal through phosphorylation of SMAD2 and SMAD3, which subsequently bind to SMAD4 and translocate to the nucleus to induce the transcription of target genes (Fig. 1). The role of this canonical SMAD-mediated pathway will be evoked later in this manuscript. Alternatively, the TGFβ may also act through SMAD-independent pathways, including the mitogen-activated protein kinase (MAPK) pathway, the TGFβ-activated kinase (TAK)-1, the phosphatidylinositol-3 kinase (PI3K), and the transcriptional intermediary factor (TIF)-1γ, among others[10]. In cancer, TGFβ signaling plays a paradoxical role since it suppresses cell proliferation during tumor initiation, whereas it favors metastasis through EMT induction.

In pancreatic cancer, the TGFβ is believed to be the major inducer of EMT. TGFβ1 is detected immunohistochemically in 41.4% of pancreatic cancer patients and serum TGFβ overload is associated with dismal prognosis[11]. Clinicopathological analysis shows that TGFβ1 expression is significantly correlated with lymph node metastasis and the depth of invasion, indicating that TGFβ protumoral action prevails in pancreatic cancer. *In vitro*, TGFβ1 promotes EMT in the human pancreatic cancer cell line Panc-1 and enhances its invasion ability[12]. *In vivo*, targeting of downstream elements of TGFβ signaling leads to an EMT defect in pancreatic tumors developed by mice bearing an activated *Kras*$^{G12D}$ allele[13,14]. Indeed, *Kras*$^{G12D}$-expressing pancreatic tumors with deletions of DPC4/Smad4 and TIF1γ genes, which encode classical and alternative TGFβ signal transducers, present a glandular, cystic, well-differentiated phenotype with little or no metastasis.

The molecular pathways that support the protumoral effects of TGFβ in pancreatic cancer have been documented by *in vitro* experimentation using human pancreatic cancer cell lines. Experiments using Panc-1 cells demonstrated the implication of the Ras-dependent signal transduction pathway on the establishment of TGFβ-induced gene transcription associated with EMT. Expression of a dominant- negative

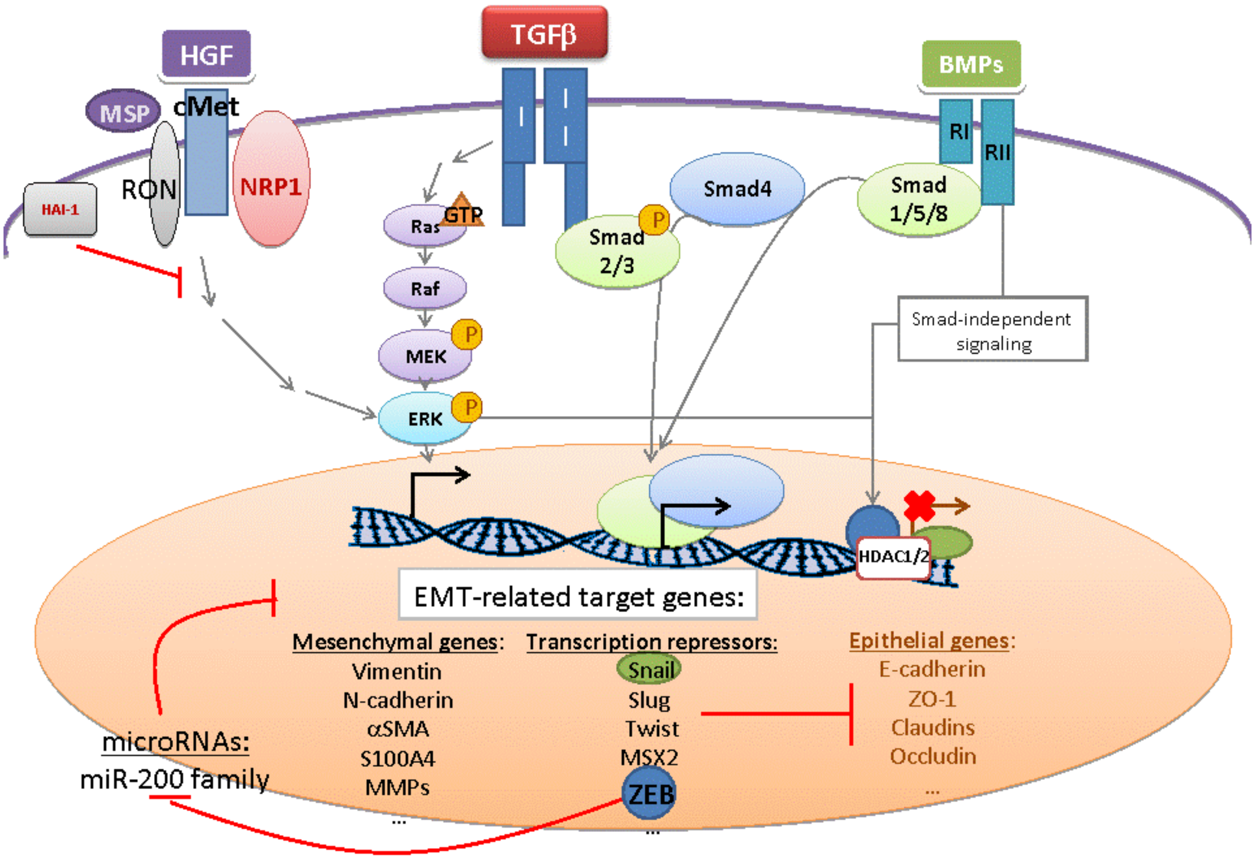

**FIGURE 1.** Growth factor–induced EMT signaling pathways in pancreatic cancer. TGFβ, hepatocyte growth factor (HGF), and bone morphogenic proteins (BMP) 2, 4, and 7 through binding of their receptors (TGFRI/RII, c-Met, and BMPRI/RII, respectively) trigger the activation of the expression of EMT-related genes, including mesenchymal genes and transcription repressors that, in turn, down-regulate the expression of epithelial genes in collaboration with histone deacetylases (HDAC)1/2. Transcription activation can be both Smads-dependent and -independent, through the activation of the Ras/ERK pathway.

$Hras^{S17N}$ mutant in these cells abolishes the EMT-related transcriptional response upon TGFβ, whereas expression of a constitutively activated $Kras^{G12V}$ mutant by itself triggers EMT-related gene profile[15]. Moreover, the selective MEK1 inhibitor PD 98059, which inhibits extracellular signal-regulated kinases (ERK)-1 and -2 activity, inhibits the induction of the EMT-related gene profile and morphological changes upon TGFβ treatment in Panc-1, and in the Colo-357 and IMIM-PC1 cell lines[12]. In these cells, TGFβ stimulation results in increased tumor cell migration, invasion, and scattering associated with moderate, but sustained, activation of ERK2. Altogether, these data indicate that the Kras-mediated activation MEK-ERK–signaling pathway is essential for TGFβ-induced EMT in pancreatic cancer cells.

In addition, a study from Gordon et al. using both Panc-1 cells and human adenocarcinoma specimens demonstrated a down-regulation of the type III TGFβ receptor (TβRIII) during pancreatic cancer cell EMT that suggested a negative regulatory role for this TGFβ coreceptor on EMT[16]. Indeed, the authors found that TβRIII loss was necessary for increasing cell motility and invasiveness of Panc-1 cells resulting from TGFβ-induced EMT. TβRIII expression on the cell surface is specifically lost after treatment of Panc-1 cells with TGFβ due to increased shedding of this receptor. In human pancreatic cancers, TβRIII expression is inversely correlated to the malignancy grade. Therefore, it was proposed that loss of TβRIII expression is a key event during EMT in pancreatic cancer cells[16].

## Hepatocyte Growth Factor (HGF)

Both HGF and its receptor c-Met are overexpressed in pancreatic adenocarcinomas[17]. HGF treatment promotes cell invasiveness of the pancreatic cancer cell line Colo-357 through c-Met signaling that involves its association with the neuropilin-1/NRP1[18]. The HGF activator inhibitor-1 (HAI-1), encoded by the serine protease inhibitor Kunitz type 1 (SPINT1) gene, is a membrane-associated protease inhibitor. Stable knock-down of HAI-1 in the human pancreatic cancer cell line SUIT-2 induces EMT, along with induction of the Smad-interacting protein 1 (SIP1/ZEB2), an E-cadherin transcriptional repressor. Conversely, the overexpression of HAI-1 in metastatic pancreatic cancer cells leads to restoration of E-cadherin levels and epithelial morphology *in vitro*[19].

RON (also known as MSTR1), a tyrosine kinase receptor related to c-Met, is overexpressed in 93% of human pancreatic cancers and its expression is detected in all human pancreatic cancer cell lines tested[20]. *In vitro* experiments using the pancreatic cancer L3.6pl cell line treated with the RON ligand, macrophage stimulating protein (MSP/MST1), established the implication of RON in EMT triggering in pancreatic cancer cells. MSP treatment increases ERK phosphorylation (Fig. 1), cell migration, and invasiveness in L3.6pl cells. Conversely, concomitant incubation with a RON-neutralizing monoclonal antibody (MoAb) inhibits L3.6pl cell migration and invasion. Furthermore, RON stimulation by MSP leads to a decrease in E-cadherin expression and nuclear translocation of β-catenin in these cells. The promoting effect of RON on cancer cell invasiveness was confirmed *in vivo*, since RON MoAb severely inhibited subcutaneous and orthotopic tumor growth in nude mice. Therefore, both RON and c-Met can be considered as potential therapeutic targets against HGF-induced pancreatic cancer cell invasiveness.

## Bone Morphogenetic Proteins (BMPs)

BMPs and the BMP-signaling pathway play important roles in pancreatic cancer cells. BMP family members BMP-2, BMP-4, and BMP-7 were shown to induce EMT in the Panc-1 cell line since they trigger the acquisition of EMT classical hallmarks, such as E-cadherin loss and enhanced migration and invasiveness. BMP-mediated invasiveness of Panc-1 cells is partly due to increased expression and activity of matrix metalloproteinase (MMP)-2[21]. Moreover, BMP reduces expression of the inhibitory TGFβ type III receptor (TβRIII) during pancreatic cancer progression. Smad1 is required for BMP-induced loss of TβRIII expression and invasiveness, and partially responsible for BMP-mediated MMP-2 up-regulation. Finally, BMP4 treatment induces homeobox gene MSX2 expression, which is associated with EMT in pancreatic cancer cells. Silencing of MSX2 abolishes BMP-mediated EMT. Therefore, BMP4 promotes pancreatic cancer progression by supporting EMT through MSX2 induction.

## Vascular Endothelial Growth Factors (VEGF)

VEGF-α and -β, through their receptor VEGFR1, were shown to induce cell migration, invasion, and EMT of pancreatic cancer cells. Treatment of L3.6pl cells with VEGF proteins leads to acquisition of spindle-like morphology, loss of cell polarity, and the development of pseudopodia[22]. VEGFR1 cross-linking enhances cell rearrangement of E-cadherin and β-catenin from the cell membrane to the cytoplasm and nucleus, respectively. Moreover, VEGFR1 activation leads to down-regulation of the expression of E-cadherin and plakoglobin, and to up-regulation of mesenchymal vimentin and N-cadherin. This is associated with an increase in the expression of the EMT-associated transcription repressors Snail, Twist, and Slug. Altogether, these observations indicate that VEGF is able to trigger invasion and migration of pancreatic cancer cells through VEGFR1.

## EMT-RELATED SIGNALING IN PANCREATIC CANCER

### The SMAD/STAT3 Pathway

Thus far, the Smad pathway was the most-studied TGFβ downstream signaling cascade. The Smad protein superfamily is composed of the receptor-regulated Smad1, 2, 3, 5, and 8; the co-Smad Smad4; and the inhibitory Smad6 and 7. Upon TGFβ binding to its receptor, Smad2 and 3 are activated through phosphorylation of their SSXS C-terminal motif. Subsequently, they form homo- and heteromeric complexes with Smad4 and are translocated to the nucleus to ultimately regulate the transcription of target genes. Contradictory *in vitro* data made the role of Smads in EMT controversial until *in vivo* targeting of Smad4 shed light on its pivotal role in pancreatic cancer EMT. Inactivation of the DPC4 gene that encodes Smad4 suppresses TGFβ-mediated EMT and invasiveness of pancreatic cancer cells in $Kras^{G12D}Ink4a^{KO}$ mice[23]. Blockade of EMT in Smad4-deficient pancreatic tumors is characterized by epithelial morphology and organization, persistence of epithelial markers (such as cytokeratin 19), and lack of mesenchymal markers (such as vimentin) in tumor cells. Nevertheless, Smads seem to interplay with other TGFβ signaling pathways to induce EMT in pancreatic cancer, and could even participate in negative regulation of the process. Using isogenically matched pancreatic cancer cells that differed only in the expression of Smad4, Zhao et al. demonstrated that although Smad4 is necessary for TGFβ-mediated down-regulation of E-cadherin and β-catenin and for vimentin induction, Smad4 wild-type cells present reduced invasion and metastasis in an orthotopic model of pancreatic cancer[24]. This observation may be explained by a TGFβ-mediated inhibition of STAT3(Tyr705) phosphorylation in Smad4 wild-type cells. Consistently, overexpression of a constitutively activated form of STAT3 (STAT3-C) in these cells enhances pancreatic cancer invasion. Therefore, STAT3 activation is required for invasiveness of pancreatic cancer cells and Smad4 is a negative regulator of this STAT3 EMT-associated function. Conversely, loss of Smad4 leads to aberrant activation of STAT3 and may contribute to the switch of TGFβ from a tumor-suppressive to a tumor-promoting pathway in pancreatic cancer.

Consistent with a pivotal role of STAT3 in pancreatic cancer EMT, its downstream target LIV-1 was shown to regulate the nuclear localization of Snail, which is a master regulator of EMT[25]. LIV-1 knock-down in Panc-1 cells leads to decreased Snail nuclear translocation and E-cadherin down-regulation. Moreover, LIV-1–depleted Panc-1 cells display impaired cell motility and proliferation *in vitro*, as well as reduced tumor growth and metastasis when injected in nude mice. The effect of this STAT3 target on EMT appears to operate *in vivo*, since the expression of the LIV-1 protein is positively correlated with the presence of metastasis in pancreatic carcinoma specimens.

### The Transcription Repressors Snail, Slug, Twist, and ZEB

SNAI1/Snail, SNAI2/Slug, ZEB1/δEF1/ZFHX1A, ZEB2/SIP1/ZFHX1B, TWIST1/TWIST, and TWIST2/DERMO1 are well-known regulators of EMT in development and cancer-related EMT through their repressor activity on CDH1/E-cadherin expression. Snail and Slug belong to the Snail superfamily of zinc-finger transcriptional repressors that bind to DNA on consensus E2-box-type elements C/A(CAGGTG). ZEB1 and ZEB2, two members of the ZEB family, interact with DNA through the simultaneous binding of their zinc-finger domains to high-affinity binding sites composed of CACCT and CACCTG E-boxes, as found in the CDH1/E-cadherin promoter. TWIST1 and TWIST2 are class-II basic helix-loop-helix (bHLH) transcription factors that act as heterodimers with the E-proteins E12 and E47. In addition to their role in E-cadherin repression, each one of the above-mentioned repressors may differentially regulate a subset of epithelial-related genes, including claudins, cytokeratins, cadherins, occluding, and ZO proteins[26].

Snail expression is induced upon TGFβ stimulation in Panc-1 cells through cooperation with signaling of their endogenous active Kras allele[27]. siRNA-mediated knock-down of Kras abolishes induction of Snail expression in Panc-1 cells, but not the one of the TGFβ-Smad target Smad7, indicating that Kras cooperation was specifically needed for Snail expression. Interestingly, Smad2 and 3, but not MAPK activity, are required for TGFβ-mediated induction of Snail. Thus, Kras and TGFβ-Smad signaling cooperate in the induction of Snail in a Smad-dependent manner, but independently of phosphorylation at the linker region of R-Smads by Kras signaling.

Like Snail, ZEB2 down-regulates transcription of CDH1/E-cadherin, CLDN4, CCND1, TERT, SFRP1, ALPL, and miR-200b-200a-429 primary miRNA, and up-regulates the transcription of mesenchymal markers[9,28]. Like the above-mentioned EMT-related transcription repressors, ZEB2 expression is triggered by TGFβ, TNFα, IL-1, and hypoxia.

### Ras/ERK1/2 Pathway

Kras-activating mutations occur in virtually all human pancreatic cancers. The formal proof of Kras-activating mutations as prominent initiating events in pancreatic cancer was provided by the generation of mice bearing a transgenic $Kras^{G12D}$ allele targeted to the pancreas using the pancreas-specific elastase-1 and Pdx1 promoters[13,29]. These mice develop precancerous pancreatic intraductal neoplastic lesions (PanINs) and around 25% of them present with late development of invasive pancreatic adenocarcinoma. Combination of the Pdx1-$Kras^{G12D}$ allele with deletions of either p53 or p16/p19Ink4a lead to full development of pancreatic tumors, with a 100% penetrance and lethality within 12 weeks[23,30,31].

Several evidences indicate the implication of the Ras/ERK1/2 pathways in the mesenchymal transformation of cancer cells. First of all, oncogenic $Ras^{G12V}$ or ERK2 overexpression leads to mesenchymal transformation of MCF-10A breast cancer cells[32,33]. The interplay between Ras and ERK2 was established by the abrogation of $Ras^{G12V}$-induced EMT after ERK2 shRNA-mediated knock-down[33]. In pancreatic adenocarcinoma, there is a positive correlation between EMT and the activation of ERK in cancer cells, with poor survival of patients[34]. Moreover, the induction of the MSX2 homeobox gene by BMP4 was shown to be dependent on activation of ERK and p38 MAPK pathways in collaboration with Smad proteins[35].

## CHROMATIN-REMODELING ELEMENTS IN PANCREATIC CANCER EMT

### Histone Deacetylase (HDAC)

Consistent with the protagonist role of EMT in metastasis, highly metastatic pancreatic cancer cells derived from sequential passages of primary pancreatic cancer cells display low E-cadherin expression, which is commonly attributed to the sole intervention of Snail, Slug, and ZEB1/2. Nevertheless, *in vitro* and *in vivo* evidence has been provided for the implication of HDAC activity in E-cadherin repression in pancreatic cancer[36]. Indeed, HDAC1 and HDAC2 were shown to participate in the Snail-containing E-cadherin transcriptional repressor complex. Consistently, HDAC activity was shown to be necessary for E-cadherin repression and EMT of human pancreatic cancer cells upon TGFβ treatment. Therefore, the HDAC machinery and its regulators may be considered as potential targets for antimetastatic therapy through their effect on E-cadherin repression.

### High-Mobility Group A Protein (HMGA)

HMGA2 is a nonhistone chromatin factor, expressed in undifferentiated tissues and tumors of mesenchymal origin. Its role in EMT was first demonstrated in murine mammary cells in which HMGA2 plays an activating role on Snail, Slug, Twist, and the inhibitor of differentiation (Id)2, and a subsequent inhibitory effect on E-cadherin expression upon TGFβ[37]. A recent study by Watanabe et al. showed that HMGA2 expression is necessary for maintenance of the mesenchymal phenotype of human pancreatic cancer cells after EMT[38]. HMGA2 expression in pancreatic cancer cells necessitates the activity of the Ras/MEK pathway, whose inhibition leads to reversion of the mesenchymal phenotype to an epithelial phenotype. Consistently, HMGA2 expression is inversely correlated to E-cadherin expression in pancreatic cancer tissues.

## microRNA (miR) AND PANCREATIC CANCER EMT

MicroRNAs (miRNAs) are a class of short (18–24 nucleotides), noncoding RNAs implicated in post-translational regulation of gene expression. The classical mechanism of miRNA-mediated inhibition of gene expression involves base pairing (often imperfect) to the 3' unstranslated regions of target messenger RNAs, thereby inhibiting their translation and/or triggering their degradation. The functions reported for miRNAs are multiple and miRNA specific, and seem to depend on the target genes affected, the cell type, the tissue origin, and the differentiation stage. The first studies of the role of miRNAs during EMT were performed in Madin Darby Canine Kidney (MDCK) cells in which EMT was induced by TGFβ treatment or by overexpression of the protein tyrosine phosphatase Pez[39]. This study demonstrated that EMT is associated with the down-regulation of five members of a miRNA family, the miR-200, and the miR-205, suggesting a negative regulatory role of these miRNAs on EMT. Indeed, this conclusion was confirmed since the constitutive expression of miR-200b–200a–429 completely abolished TGFβ-induced EMT in MDCK cells. A comprehensive study of EMT in Panc-1 cells by the Brabletz group showed that, in pancreatic cancer cells, the down-regulation of miR-200 family members miR-141 and miR-200c is driven by the transcription repressor ZEB1 during EMT[28]. ZEB1 and TGFβ figure among miR-200 family target genes, thus describing a negative regulatory feedback loop.

Interestingly, recent research from the same group interrogated the parallel between ZEB1-mediated miRNA repression, EMT induction, and the stemness of pancreatic cancer cells. Indeed, EMT and stemness (the capacity to self-renew, proliferate, and differentiate) share some molecular traits and their pivotal role in motility and metastasis formation. They showed that EMT-induced ZEB1 repressor down-regulates the expression of the miR-203 and miR-183, which in turn suppress the expression of stem cell factors, such as Bmi1. Moreover, EMT appears to be crucial to stemness maintenance through induction of ZEB1 and concomitant repression of proepithelial miRNAs.

Finally, EMT-mediated regulation of the miRNA profile was associated with pancreatic cancer cell resistance to chemotherapy. A differential analysis of miRNA expression in gemcitabine-resistant (Panc-1, MiaPaCa-2, and Aspc) and -sensitive (L3.6p, BxPC3, Colo357, and HPAC) cells revealed that EMT-regulated members of the miR-200 family are down-regulated in resistant cells compared to the sensitive cells[40]. Moreover, reversal of EMT and concomitant up-regulation of miR-200 family expression by isoflavone treatment rendered resistant cells more sensitive to gemcitabine. Therefore, miRNA regulation during EMT appears to be at the basis of the protumoral attributes of EMT that are motility enhancement and drug resistance.

## ALTERNATIVE INDUCERS OF PANCREATIC CANCER CELLS EMT

### Cell Adhesion

Whereas disruption of cell-cell interactions through loss of E-cadherin expression is a key hallmark of EMT, cell adhesion to the extracellular matrix was shown to support the mesenchymal phenotype. This alternative mechanism of EMT maintenance is likely to play an important role in pancreatic cancer, considering the desmoplastic microenvironment associated with pancreatic tumors. Considerable research has been reported on cell adhesion molecules, especially regarding the cadherin family. N-cadherin expression, characteristic of the mesenchymal phenotype, correlates with neural invasion, histological type, and fibroblast growth factor (FGF)-2 expression in pancreatic cancer, and with TGFβ and vimentin expression in metastatic lesions. Collagen adhesion promotes EMT of pancreatic cancer cells by inducing N-cadherin expression and the related tumor aggressiveness *in vitro*, and metastasis in a murine model[41,42]. Two collagen receptors are involved in collagen-induced up-regulation of N-cadherin in pancreatic cancer cells. The $\alpha 2\beta 1$ integrin mediates the collagen-induced signal through the downstream focal adhesion kinase (FAK). In addition, the discoidin domain receptor (DDR)-1 transduces the collagen signal through the FAK-tyrosine kinase Pyk2. Both collagen receptor complexes need the p130 Crk as a scaffold.

Periostin functions as a cell adhesion molecule with both inhibitory and promoting effects on cell migration. In pancreatic tumors, periostin is expressed mainly in stromal cells and in particular in pancreatic stellate cells, but is also detected in cancer cells. Periostin binding in pancreatic cancer cells triggers the reversion of the mesenchymal phenotype to the epithelial phenotype, a process known as the mesenchymal-epithelial transition (MET), which suppresses invasion and metastasis. Nevertheless, high periostin concentrations promote cell migration through AKT activation[43].

### Hypoxia

Hypoxia is one of the major stresses that tumor cells face within the primary tumor. Hence, it is not surprising that hypoxia was found to trigger EMT of cancer cells of various organs, including the pancreas, for the increased migratory and invasive skills EMT confers will help to escape from the asphyxiating tumor microenvironment. The mechanism allowing hypoxia-induced EMT involves the activation of the glycogen synthase kinase-3β (GSK-3β), which induces the nuclear translocation of Snail and β-catenin. In parallel, hypoxia induces enhanced migration and invasiveness by the action of the hypoxia-inducible factor (HIF)-1α through a VEGFα-dependent mechanism[44].

## EMT AND DRUG RESISTANCE IN PANCREATIC CANCER

As previously mentioned, gemcitabine resistance has been associated with a mesenchymal phenotype in pancreatic cancer cells[40]. Conversely, isoflavone-induced MET breaks the shield of gemcitabine resistance in MiaPaCa-2, Panc-1, and Aspc cells, confirming the necessity of EMT maintenance for survival upon this drug.

Resistance of pancreatic cancer cells to 5-fluorouracil (5-FU) and cisplatin was also shown to be related to a mesenchymal genetic signature[45]. The mechanism allowing the resistance to the three drugs, gemcitabine, 5-FU, and cisplatin, seems to be common and to necessitate EMT-induced ZEB1 expression, for knock-down of this transcription repressor renders cells more sensitive to any of these drugs. Moreover, as expected, silencing of ZEB1 in drug-resistant pancreatic cell lines increases the expression of epithelial markers such as E-cadherin, EVA1, and MAL2.

In addition, Notch-2 and its ligand, Jagged-1, were shown to be overexpressed in gemcitabine-resistant pancreatic cancer cells in which they support EMT[46]. Indeed, knock-down of Notch-2 or Jagged-1 provokes a partial reversal of the mesenchymal phenotype of the gemcitabine-resistant cells, with down-regulation of vimentin, Snail, Slug, ZEB1, and nuclear factor-kappaB (NF-κB). Furthermore, down-regulation of Notch signaling reduces migration and invasion of gemcitabine-resistant cells. Therefore, regulators of EMT, such as ZEB1 and Notch-2, may maintain drug resistance in human pancreatic cancer cells and be considered as potential therapeutic targets for adjuvant strategies.

## CONCLUSION

EMT is at the basis of the most hassling features of pancreatic cancer cells, which are their great invasiveness and drug resistance. Furthermore, the molecular pathways associated with EMT support not only the metastatic potential of pancreatic cancer cells, but also tumor-promoting processes ranging from initiation to desmoplasia and cancer cell stemness. Consequently, current and future studies of pancreatic cancer EMT have revealed, and will certainly reveal, a panel of promising therapeutic targets that should be seriously considered for treatment of this so-far-incurable disease. As faithful genetically engineered mouse models of pancreatic cancer are now available, researchers are encouraged to address very important and yet-unanswered questions about pancreatic EMT. For instance, what are the molecular events or factors that permit the switch of antitumoral signals, such as TGFβ, onto EMT-inducing ones? How much does the abundant stroma of pancreatic tumors contribute to cancer cell EMT? Is EMT a cause or a consequence of the expansion of pancreatic stellate cells during pancreatic adenocarcinoma? Is the spindle-like/fibroblastic phenotype the only possible mesenchymal fate of pancreatic cancer cell EMT? Therefore, future research on pancreatic cancer EMT promises to be exciting.
